# Approach to Rectal Cancer Surgery

**DOI:** 10.1155/2012/247107

**Published:** 2012-06-25

**Authors:** Terence C. Chua, Chanel H. Chong, Winston Liauw, David L. Morris

**Affiliations:** ^1^Hepatobiliary and Surgical Oncology Unit, UNSW Department of Surgery, St George Hospital, Kogarah, NSW 2217, Australia; ^2^St George Clinical School, Faculty of Medicine, UNSW, Sydney, NSW 2052, Australia; ^3^Cancer Care Centre, Department of Medical Oncology, St George Hospital, Kogarah, NSW 2217, Australia

## Abstract

Rectal cancer is a distinct subset of colorectal cancer where specialized disease-specific management of the primary tumor is required. There have been significant developments in rectal cancer surgery at all stages of disease in particular the introduction of local excision strategies for preinvasive and early cancers, standardized total mesorectal excision for resectable cancers incorporating preoperative short- or long-course chemoradiation to the multimodality sequencing of treatment. Laparoscopic surgery is also increasingly being adopted as the standard rectal cancer surgery approach following expertise of colorectal surgeons in minimally invasive surgery gained from laparoscopic colon resections. In locally advanced and metastatic disease, combining chemoradiation with radical surgery may achieve total eradication of disease and disease control in the pelvis. Evidence for resection of metastases to the liver and lung have been extensively reported in the literature. The role of cytoreductive surgery and hyperthermic intraperitoneal chemotherapy for peritoneal metastases is showing promise in achieving locoregional control of peritoneal dissemination. This paper summarizes the recent developments in approaches to rectal cancer surgery at all these time points of the disease natural history.

## 1. Introduction

Rectal cancer is often camouflaged to be part of colorectal cancer with Australian statistics approximating 14 225 cases of bowel cancer in 2008 with a higher predominance among men and those above 50 years of age [1]. Meanwhile, the American counterpart recorded rectal and colon cancer to be the third leading malignancy plaguing both sexes in 2012 whereby 9% of deaths in each gender group was attributed to the disease [2]. The risk of death before age 75 has declined over the years from a high of 1 in 50 in 1987 to 1 in 91 in 2007 and this is attributed to better early detection of precancerous lesions and management of the disease [1, 2].

Treatment of rectal cancer is primarily surgical and is highly dependent on the preoperative staging [3]. Early cancers, especially T1 tumors, have been controversially proposed for local excision either via transanal excision or transanal endoscopic microsurgery (TEMS) [4–6]. While some report good outcomes measurable to radical surgery, others beg to differ prompting the need for salvage therapy [4–7]. Later stages of cancer usually follow the traditional approach of abdominoperineal resection (APR) or anterior resection and its success at reducing local recurrence is improved by total mesorectal excision (TME) [8, 9]. However, these procedures are associated with their own set of morbidity and mortality [10, 11]. Other therapeutic modalities such as chemotherapy and radiotherapy have been included in conjunction with surgery particularly in advanced cancers. They can be used preoperatively or postoperatively either to increase the chances of a sphincter-preserving procedure to allow better quality of life or with a curative intent [12–14]. An overall cancer-specific 5-year survival of 77% has been reported [15].

In this paper, we seek to discuss the modern approach to rectal cancer surgery at all disease time points from preinvasive and early rectal cancer, resectable rectal cancer, and locally advanced and metastatic rectal cancer with an emphasis on presenting some of the controversies and the accepted standards of treatment.

### 1.1. Local Excision of Preinvasive and Early Rectal Cancer

The advent of endoscopic mucosal resection (EMR) for rectal tumours could not have been timelier as the race to provide a treatment that is highly efficacious and of low morbidity continues. EMR has been in practice for a few decades now and is commonly used to address gastrointestinal pathologies such as a Barrett's oesophagus, and early gastric cancers (EGC) with a reported local recurrence rate of 2% in EGC [16–18]. The procedure focuses on removal of diseased mucosal tissue or at most, the superficial submucosa instead of a full-thickness excision [16, 19]. It can be done by means of a strip biopsy, local injection of hypertonic saline-epinephrine in the “inject, lift and cut” technique, “cup and suction” otherwise known as the EMR-cap technique or EMR with band ligation. If necessary, more than one of the aforementioned methods can be applied [16, 20, 21]. 

EMR is an alternative to the locally-approached transanal excision (TAE) and transanal endoscopic microsurgery (TEMS). Generally, local excision of rectal cancer is indicated for early disease that encompasses its precursor (dysplasia or adenoma), T1 tumours that display moderate-to-well differentiation and no lymphatic or vascular invasion [6, 16, 17, 22]. At present, it is known that EMR can be performed on flat, sessile, or lateral spreading rectal tumours [22–24]. The success of EMR is reflected through its high overall cure rate with some reporting 91% for treatment-naïve patients and others at 96% [23, 25]. 

One of the common consequences of EMR is bleeding which occurs from 1%–45% of cases [26]. The bleed can be minor and is easily manageable with haemostatic clips or severe requiring blood transfusions or even surgery [19, 24, 26]. 0.7% to 4% of cases are complicated with perforation which can appear later as abdominal distension and pain necessitating further operation [25, 26]. Other adverse events also include postprocedural abdominal pain and serositis [25].

While EMR is an excellent therapeutic option for rectal tumours, it is operator dependent. Identifying the lesion especially if it is a lateral spreading tumour can be challenging to the untrained eye as it displays subtle changes but this is improved by a dye-staining technique [22]. A meta-analysis showed that the chances of a complete en bloc resection is directly proportional to experiences if the operator [27]. Removal of diseased-tissue greater than 2 cm requires a piecemeal resection. The concern is that complete resection and a proper histological evaluation is rendered difficult with larger lesions, thus running a risk of local recurrence [23, 25, 28]. In spite of this, some studies showed that the success of the resection is not statistically different between the two techniques [26, 29]. 

Complicating the procedure further is the second attempt of EMR for recurrent lesions. Failure of the mucosa to lift during the “inject, lift and cut” technique may occur secondary to submucosal fibrosis. The success rate of a repeated resection is reduced from 91.0% in a treatment-naïve patient to 74.5% in a previously attempted lesion [25]. It is reported that the time for conversion of a precursor lesion to cancer is variable, with dwelling times in the adenoma state ranging from 4 years to as long as 48 years [30]. In fact, flat adenomas need not necessarily be treated as it is almost always noninvasive [22]. This questions the need for EMR in such lesions and more so, the implication on the patient who is no doubt heading for months of unwarranted anxiety.

Nevertheless, EMR is a safe and effective treatment modality that is minimally invasive and only requires conscious sedation [25, 26, 28]. It is also efficient with Moss et al. reporting a mean procedural duration of 25 minutes while another study accounted a range of 25–137 minutes [25, 29]. When possible, patients can be treated as an outpatient with discharges within the same day [31, 32]. A comparison between TAE and endoscopic resection showed that the latter was associated with a significantly shorter hospital stay of 2.7 ± 1.1 days with a mean difference of 6.2 days [19]. Similarly, TEMS recorded a range of 0–44 days of hospitalization while EMR had a range of 0–27 days [31]. EMR per se is not as costly as the other procedures available, thus allowing a cheaper and less invasive option for the patient. This translates into reduced expenditure for the spender [20, 29, 33].

On the other hand, TEMS and TAE are not as inferior as one might think. Lesions larger than 8 cm in diameter can be removed with good effect via TEMS [34, 35]. Where EMR may fail to completely remove the tumour, both TEMS and TAE can compensate for it [31, 36]. First attempt of TEMS for large rectal adenoma is reported to have a lower early local recurrence rate at 10.2% compared to EMR at 31.0% (*P* < 0.001) [31]. Meanwhile, low risk-T1 tumour recurrence rate following TEMS is 0–10% while Santos et al. reported a 12% recurrence rate following EMR [28, 37]. TAE is estimated to have a 15% local recurrence rate of the malignancy at five years [38]. It would seem that the recurrence rate following TAE is high compared to EMR but contradicting this is a 32-patient study by Lee et al. which showed no recurrences of cancer following either endoscopic resection or TAE at a median followup of 15 months (range 6–99) [19]. Studies evaluating the disease-specific and overall survival rates (TEMS versus radical surgery and TAE versus radical surgery) showed that the values were not statistically significant to one another indicating the efficacy of these procedures [5, 38].

EMR appears to be favourable as TEMS and TAE have the added adverse effects including wound breakdown, incontinence, urgency, strictures, neuralgia, and anovaginal fistula [34, 35, 37, 39]. Barendse et al. studied both EMR and TEMS and discovered that the former was associated with half the percentage (12%) of postoperative complications compared to the latter (24%) [31]. However, given the variable recurrence rates, there is some apprehension towards EMR. In view of this, no accurate decision can really be made. Instead, it is a judgment call by the surgeon and more importantly the patient himself based on the local expertise and the pros and cons of each option available. As we push the fort of combination treatment, it is likely that in the future, local excisional strategies will become a more commonly adopted strategy in complete or subcomplete responding tumors after chemoradiotherapy. However, the limitation of mesorectal sampling may mean that more intensive followup is required to detect recurrence at the local excisional site and/or mesorectal lymph nodes.

### 1.2. Standardized Surgery in the Era of a Multimodality Approach for Resectable Rectal Cancer

Rectal cancer differs from colon cancer whose risk of local recurrence is low. Its proximity to the anal sphincter also makes this a major consideration into the surgical approach towards resection. In the localized setting, a multimodality approach has in recent years been developed and investigated in trials to improve local recurrence, disease-free and overall survival using a variety of sequences of chemotherapy and radiotherapy. Prior to surgery, adequate local staging is paramount in the surgical planning and accurate prediction of the extent of bowel wall involvement may be obtained through endorectal ultrasonography and magnetic resonance imaging may serve the similar purpose but also identify the involvement of lymph node metastases [40]. The aims of rectal cancer surgery are to remove the tumor with an adequate distal margin of a minimum of 2 cm in the case of a low rectal tumor with sphincter preservation or 5 cm in the case of a rectosigmoid/upper rectal tumor with restoration of intestinal continuity through an anastomosis. This operation is known as anterior resection. If a 2 cm distal margin cannot be secured, an abdominoperineal excision with enbloc resection of the entire anorectum and an end colostomy is required. Surgery should be performed using a “no-touch” technique with high ligation of the inferior mesenteric artery to achieve adequate lymphatic sampling through harvesting of the sigmoid mesentery and mesorectum. Tumors of the middle and lower rectum require a total mesorectal excision (TME) [41]. TME reduces the risk of local recurrence and although a prospective randomized trial has not been conducted to verify its efficacy, longitudinal data derived from The Netherlands where a TME trial was conducted following rigorous training of colorectal surgeons demonstrated that there was an observed reduction in rate of local recurrence from 16% to 9% with TME surgery being an independent predictor of overall survival [42]. Further, data from the MRC CR07 and NCIC-CTG CO16 randomized trials demonstrated that the plane of surgery achieved in patients undergoing rectal cancer surgery impacted on local recurrences with a 3-year local recurrence rate of 4% for patients whose surgery was completed with achievement of the mesorectal plane, 7% for intramesorectal plane and 13% for muscularis propria plane [43]. Pertinent also in low rectal cancers requiring abdominoperineal excision, to avoid a “coning effect” in the deep pelvis as the tumor is approached from both the abdomen and perineum, extended abdominoperineal excision incorporating resection of the levator muscles to reduce inadvertent bowel perforation and breaching of the circumferential resection margin [44].

#### 1.2.1. Laparoscopic or Open Surgery

Today, in the current era of laparoscopic surgery where shorter postoperative hospital stays, reduction in pain scores, shorter time of return of bowel function, lower treatment cost, and improved cosmesis may be achieved, these standardized surgical resections have been demonstrated to be feasible in the laparoscopic approach [45]. Initially, the laparoscopic approach was first examined in colon cancer with at least 4 large randomized trials; COST Study Group trial from the USA, COLOR trial from Europe, MRC CLASICC trial from the UK, and a trial in rectosigmoid cancers from the Prince of Wales Hospital in Hong Kong demonstrating equivalent oncologic efficacy with similar overall survival, disease-free survival and local and distant recurrences [46–49]. These studies although predominantly examined in the setting of colon cancer were quick to translate into standard practice for rectal cancer despite limited large scale prospective randomized trials. However, there remain concerns over the ability to achieve adequate mesorectal excision and clear surgical margins in laparoscopic rectal cancer surgery. In the UK MRC CLASICC trial, there was a 34% conversion rate from laparoscopic to open surgery in the rectal cohort, increase performance of TME surgery to ensure adequacy of the distal resection margin in the laparoscopic group because of the inability to palpate the tumor for localization. Nonetheless, there was no difference in positive circumferential resection margin, local recurrence, disease-free, and overall survival in both the laparoscopic versus open anterior resection and abdominoperineal resection groups [48].

The Prince of Wales Hospital group from Hong Kong reported a small prospective randomized trial of laparoscopic assisted versus open abdominoperineal resection for low rectal cancer randomizing 99 patients (51 lap-assisted and 48 open) demonstrating earlier return of bowel function, decrease time to mobilization, lesser analgesia requirement, longer operative time, and higher direct cost in the lap-assisted group without difference in morbidity and mortality [50]. In an update of this trial, after 10 years of followup, the authors reported higher rates of bowel obstruction requiring hospitalization and intervention in the open group but similar oncologic outcomes were shown with 10-year survival of 83.5% and 78% (*P* = 0.595) and 10-year disease-free survival 82.9% and 80.4% (*P* = 0.698) in the lap-assisted and open group, respectively [51]. In the COREAN trial that randomized 170 patients in each arm to laparoscopic and open surgery for mid or low rectal cancer after preoperative chemoradiotherapy, the laparoscopic group had lower amount of blood loss, longer operative time, quicker recovery of bowel function, and a lesser amount of analgesic requirement. Surgical quality indicators including the circumferential resection margin, macroscopic quality of the TME specimen, number of harvested lymph nodes, and perioperative morbidity were similar between groups [52]. In a Spanish randomized trial of 204 patients of whom 78.6% in the open group and 76.2% in the laparoscopic group underwent sphincter-preserving surgery; blood loss was greater in the open surgery group, operative time was longer in the laparoscopic group, and return to diet and hospital stay was longer in the open surgery group. Complication rates were similar between groups but a larger number of lymph nodes were isolated in the laparoscopic group [53]. Together, these three small randomized trials suggest that the laparoscopic approach achieves improved short-term outcomes without compromising the surgical quality of rectal cancer operations in skilled hands but a longer operative time is required. Longer followup of these trials and results of ongoing larger trials will confirm the long-term oncologic outcomes of laparoscopic rectal cancer surgery.

#### 1.2.2. Sequencing of Multimodality Therapy

Thorough preoperative clinical staging is paramount in the sequencing of multimodality therapy for rectal cancer. For smaller tumors T1/T2, surgery alone with wide surgical resection of low anterior resection or abdominoperineal resection for distal lesions not amendable to low anterior resection may be performed. This allows sampling of mesorectal lymph node for accurate pathological staging. In patients who are medically unfit or who adamantly refuse to undergo standardized resectional surgery, local excision with or without chemoradiotherapy may be considered an option to palliate early-stage disease. This strategy fails to sample the mesorectal lymph nodes that are essential in disease staging. In a large single institution cohort study comparing their retrospective experience of 350 with stage I rectal cancer of whom 283 patients (80.9%) underwent standardized resection and 67 patients (19.1%) undergoing local excision, 5-year local recurrence was 14.1% in the local excision group compared to 3.3% in the standardized resection group [54]. This significantly higher local recurrence rate may then be salvaged through multimodality approach combining preoperative chemoradiotherapy and surgery. However, at times, these local recurrences may not be resected with standardized resectional surgery and may require radical surgeries such as a pelvic exenteration.

For clinically staged T3/T4 rectal tumors without clinically identified nodal disease (stage II) who undergo TME surgery with either a low anterior resection or abdominoperineal resection with harvesting of at least 12 lymph nodes examined and staged as pN0 chemoradiation is not required. Chemoradiation may be considered in the setting of pT3N0 tumors with adverse pathologic features, non-TME surgery or in those with fewer than 12 lymph nodes harvested. For T3/T4 tumors with lymph node metastases (stage III) identified clinically, treatment involves both chemoradiation with fluorouracil (5-FU) and total mesorectal excision (TME) based surgery. However, there remain enormous controversies regarding the optimal sequence of these therapies. Postoperative chemoradiation was shown to achieve superior results over postoperative radiation alone with a 34% reduction in recurrence rate with reductions observed for local recurrences and distant metastasis [55]. When postoperative chemotherapy was compared to radiotherapy in the NSABP R-01 randomized trial, postoperative chemotherapy appeared to improve survival and radiotherapy reduced the incidence of locoregional recurrence without survival improvements [56]. Given the benefits of chemoradiation in achieving local control as an adjunct to surgery, the German Rectal Cancer Study Group then conducted a randomized trial of 421 patients to determine the perisurgical sequencing of chemoradiation for rectal cancer. These investigators compared preoperative to postoperative chemoradiation and demonstrated that the 5-year cumulative incidence of local recurrence was 6% in the preoperative arm compared to 13% in the postoperative arm (*P* = 0.006) with fewer grad 3/4 acute and long-term toxic effects of chemoradiation observed in the preoperative arm compared to the postoperative arm [57]. In a smaller Korean trial, the improved effects of local control was not demonstrated in the preoperative compared to the postoperative chemoradiation arm, however, it was shown that an increased rate of sphincter preservative surgery could be achieved [58]. The exact type of preoperative therapy was also recently debated with a short course radiation (25 Gy in 5 fractions) followed by surgery a week after or a long course chemoradiation (50.4 Gy in 28 fractions combined with systemic chemotherapy) followed by surgery four to six weeks after. The brief use of radiotherapy in the short course setting has been argued upon its role in providing adequate tumor response to allow sphincter preservative surgery. Two randomized trials; Polish (*n* = 312) and Australian (*n* = 326) compared these two regimens and both trials showed a lower rate of early acute toxicity and reduce cost of treatment without any difference in long-term oncologic outcomes [59, 60].

After preoperative chemoradiation, guidelines recommend adjuvant chemotherapy for patients with node positive disease. However, the EORTC 22921 trial of 785 clinically staged T3/T4 rectal cancer patients randomized to receive adjuvant fluorouracil based chemotherapy after preoperative (chemo) radiotherapy and surgery showed no survival benefit of chemotherapy on disease-free survival. However, specific subgroup analysis was performed to determine the appropriate role of adjuvant chemotherapy and showed that pathologically staged T0-2 (ypT0-2) patients appeared to benefit in terms of both disease-free and overall survival from adjuvant chemotherapy compared to ypT3/T4 patients. Importantly, adjuvant chemotherapy did not appear to demonstrate any difference in outcomes of patients with ypN0 or ypN+ disease. This demonstrates that further benefits of adjuvant chemotherapy are only observed in responding patients (ypT0-2) [61]. Further trials are required in this area to determine the appropriate role of adjuvant chemotherapy and the selection of high-risk populations who may then benefit from other modern adjuvant agents.

## 2. Role of Surgery for Advanced and Metastatic Rectal Cancer

### 2.1. Locally Advanced or Local Recurrence

Tumors extending beyond the rectal wall with invasion into surrounding viscera are considered locally advanced rectal cancer. Often in patients who develop local recurrence, recurrent disease often similarly involve adjacent structures where the previously excised rectal tumor was located. Although its incidence has decreased following total mesorectal excision and the incorporation of preoperative chemoradiation, when it occurs, it remains a debilitating condition that is difficult to treat. Palliative radiotherapy may provide brief symptom relief for an average of 3 months with median survival in these patients being between 12 and 24 months [62–64]. Surgery may provide a long-term palliation to the debilitating symptoms of pelvic recurrences. In the curative setting, delivery of long course preoperative chemoradiotherapy may induce tumor down-staging to facilitate surgical resection. In a randomized trial comparing neoadjuvant radiotherapy to chemoradiotherapy in 207 patients with locally unresectable T4 primary rectal or local recurrent rectal cancer, chemoradiotherapy compared to radiotherapy facilitated higher potential for an R0 resection (84% versus 68%; *P* = 0.009), improved local control in patients who underwent a R0 or R1 resection (82% versus 67% at 5 years; *P* = 0.03), improved time to treatment failure (63% versus 44%; *P* = 0.003), cancer-specific survival (72% versus 55%; *P* = 0.02) and overall survival (66% versus 53%; *P* = 0.09) [65]. The surgery involved necessitated pelvic exenteration where adjacent organs are resected with an aim to achieve clear margins. In patients with primary T4 rectal cancer and recurrent rectal cancer, 28% and 20% of patients in the chemoradiotherapy arm and 27% and 46% in the radiotherapy arm, respectively, required exenteration. Intraoperative radiotherapy (external-beam) (IORT) is another approach to improve local control. This treatment modality has been investigated in patients with locally advanced unresectable rectal cancer after chemoradiotherapy down-staging and surgery. Its application is best used in the setting of a complete resection. Valentini et al. reported 100 patients with T4M0 tumors undergoing R0 resection after down-staging by chemoradiotherapy and showed that 5-year local control was 90% in patients with R0 surgery and 100% in patients with R0 surgery and IORT. Further, IORT did not appear to compensate for suboptimal surgery with 5-year overall survival of 68% observed in patients with R0 surgery compared to 22% in R1 or R2 surgery [66]. Such radical surgery however is not widely performed and only available in specialized institutions. In a pattern of care study of the United States population through data identified from the Surveillance, Epidemiology and End Results (SEER) registry, only 33% of patients with locally advanced adherent colorectal cancer underwent multivisceral resection for which was shown to be associated with improved overall survival [67]. 

### 2.2. Liver Metastases

In patients with synchronous rectal cancer with liver metastases, there remains an enigma over the appropriate sequencing of chemotherapy, radiotherapy, and surgery. In a study from the Erasmus University, van der Pool et al. reported a consecutive series of 57 patients of whom 29 patients underwent resection of the primary tumor first, 8 patients underwent simultaneous rectal and liver resection and 20 underwent a liver-first approach achieving a median survival of 47 months and 5-year survival of 38%. This was achieved in a multidisciplinary setting where an individualized approach towards treatment was taken. In general, if resection of both primary tumor and liver metastases may be completed in one surgery, this approach may be favored. If the liver metastases are not completely resectable during the rectal surgery or are too advanced for hepatectomy irrespective, neoadjuvant chemotherapy is preferred followed by a liver-first approach followed by restaging and preoperative radiotherapy and rectal surgery. In patients with metachronous liver metastases, the evidence of hepatectomy is based on current literature available for colorectal liver metastases. Results of large clinical series have shown that median survival range from 43 months to 64 months with 5-year survival ranging between 37% to 51% [68, 69]. Of note, in a large international multi-institutional registry study, rectal primary tumor were associated with extrahepatic recurrences after hepatectomy for liver metastases, hence emphasizing the importance of local control in the pelvis [69]. However, there is no difference in colon or rectal based primary tumor site impacting outcomes after hepatectomy for colorectal liver metastases [70]. 

### 2.3. Lung Metastases

Epidemiological and observatory data from the followup of patients with curatively treated colorectal cancer have shown that rectal cancer patients have a higher preponderance to developing recurrence in the lungs [71–73]. In a large population based study of 30 years in Burgundy (France), Mitry et al. reported that lung metastases often accompanied liver metastases with synchronous lung metastases being more common in left colonic and rectal cancers [71–73]. Surgery for lung metastases is indicated if the lung metastases are the only site of disease and a complete resection may be achieved. Where extrapulmonary metastases are present, a highly selective approach should be taken often after adequate tumor response to systemic chemotherapy to select patients whose disease is amendable to resection of both lung and extra-pulmonary metastases. The highest level of evidence for resection of lung metastases comes from a large systematic review of 20 published studies for pulmonary metastasectomy for colorectal lung metastases. Pfannschmidt et al. report a median 5-year overall survival of 40% in this selected group of patients who underwent surgery [74]. Site of the primary tumor did not result in different survival outcome. However, given that the liver will often be involved when there is lung metastases, it is important that the selection of patients for pulmonary metastasectomy should include a sufficient disease-free interval from previous liver resection, use of prethoracotomy CEA levels and the absence of mediastinal lymph node involvement as a separate selection criteria in this group of patients. Presently, a randomized trial (PulMiCC) funded by Cancer Research UK is seeking to investigate if pulmonary metastasectomy contributes to improved survival of patients with colorectal lung metastases by randomizing patients with a history of resected colorectal cancer who are found to have pulmonary metastases to be randomly allocated to “active monitoring” or “active monitoring with pulmonary metastasectomy” with overall survival, relapse-free survival, lung function, and patient-reported quality of life as endpoints of this clinical trial [75].

### 2.4. Peritoneal Metastases

Shedding of tumor during the difficult abdominoperineal resection, low anterior resection operation, or invasion of a large T3/T4 tumor in the upper rectum above the peritoneal reflection may result in the shedding of free peritoneal tumor cells within the abdominopelvic peritoneal cavity. The growth and implantation of these cells may result in the development of peritoneal metastases (carcinomatosis). Results of cytoreductive surgery and hyperthermic intraperitoneal chemotherapy (HIPEC) have shown that this combined modality technique allows complete excision of peritoneal tumors with locoregional control achieved through the chemoperfusate. In colorectal cancer, a randomized trial comparing cytoreductive surgery and HIPEC demonstrated a median survival of 22.3 months compared to 12.6 months in patients receive systemic chemotherapy with or without palliative surgery [76]. The two-fold survival benefit provides evidence of its efficacy. However, in this trial of 105 patients, only 12 patients had rectal cancer. Again, in a large registry study of the French experience of HIPEC in colorectal cancer, of 523 patients included, only 36 patients (7%) had primary colorectal tumor of rectal origin [77]. In another international registry of 506 patients with colorectal peritoneal metastases undergoing cytoreductive surgery and HIPEC, there were 40 patients (8%) with the primary tumor of rectal origin and the median survival of these patients was 19.2 months compared to 24 months in patients with tumors of sigmoid origin, 17 months for patients with tumors of the right colon and 20 months for patients with tumors of the left colon [78]. These results inform us that peritoneal metastases from rectal cancer is less common but prevent us from drawing any meaningful conclusion on whether there may be disparate survival outcomes for colon and rectal cancer patients with peritoneal metastases. Based on the currently available evidence, selected rectal cancer patients with limited peritoneal disease burden may be considered for cytoreductive surgery and HIPEC.

## 3. Conclusion

Rectal cancer surgery has made significant advancement at all-time points of the natural history of this disease. There are now minimally invasive local excision options that are currently being tested for efficacy as we await further clinical trials to verify its efficacy for pre-invasive and early lesions. Laparoscopic rectal cancer surgery incorporating total mesorectal excision is now emerging as the standard surgical approach with ongoing clinical trials that will confirm its short and long-term oncologic efficacy. There is now evidence based results from clinical trials for both preoperative short and long-course chemoradiation prior to surgery for resectable tumors for which has been shown to improve local control of disease albeit its aim achieving sphincter preservative surgery where possible. Ultimately, the goal of this being to achieve adequate sampling of mesorectal lymph nodes to provide adequate information for tumor staging and prediction of local and distant recurrences for which will guide treatment decisions. There is now evidence for resecting metastases from rectal cancer from local recurrences, liver, lung, and peritoneal metastases based on a body of retrospective clinical data with long-term followup.
